# Sedentary lifestyle induces oxidative stress and atrophy in rat skeletal muscle

**DOI:** 10.1113/EP092331

**Published:** 2025-01-29

**Authors:** Irem Gungor‐Orhan, Senay Akin, Scott K. Powers, Seda Olgaz‐Bingol, Haydar A. Demirel

**Affiliations:** ^1^ Department of Exercise and Sport Sciences, Exercise and Sport Physiology Division, Faculty of Sport Sciences Hacettepe University Ankara Türkiye; ^2^ Department of Applied Physiology and Kinesiology University of Florida Gainesville Florida USA; ^3^ Turkish Doping Control Center Hacettepe University Ankara Türkiye; ^4^ Faculty of Sport Sciences Near East University Nicosia Cyprus

**Keywords:** 4‐HNE, antioxidant enzymes, AOPPs, bed rest, soleus

## Abstract

Abundant evidence indicates that skeletal muscle plays a key role in regulating metabolic homeostasis. Therefore, maintaining healthy skeletal muscles is essential to good health. While prolonged muscle inactivity is known to cause oxidative stress and muscle loss, it remains unclear whether a shift from an active to a sedentary lifestyle induces similar effects. This study tested the hypothesis that transitioning to a sedentary lifestyle rapidly leads to oxidative stress and muscle loss in the load‐bearing soleus muscle. Adult Wistar rats were randomly divided into control (CON; *n* = 8) and sedentary (SED; *n* = 8) groups. During a 7‐day experimental period, CON rats were housed in standard cages allowing free movement, while SED rats were confined to smaller cages promoting sedentary behaviour. Soleus muscles were analysed for antioxidant enzyme activities (superoxide dismutase (SOD), catalase (CAT) and glutathione peroxidase (GPX)), as well as two oxidative stress biomarkers (advanced protein oxidation products (AOPPs) and 4‐hydroxynonenal (4‐HNE)). Sedentary behaviour caused a 17.2% reduction in the soleus‐to‐body weight ratio (*P *< 0.001). Moreover, the activities of SOD, CAT and GPX were significantly lower in the soleus muscle of SED animals (*P *< 0.05), while AOPPs and 4‐HNE levels were higher (*P *< 0.001 and *P *< 0.05) compared to CON animals. These findings provide the first evidence that transitioning from an active to a sedentary lifestyle leads to the rapid onset of oxidative stress and atrophy in the soleus muscle. Importantly, the results suggest that impaired antioxidant defences contribute to sedentary behaviour‐induced oxidative stress in load‐bearing muscles.

## Abstract (highlights)

- **What is the central question of this study?**
  Long‐term skeletal muscle inactivity causes muscle atrophy: Does transitioning from an active to a sedentary lifestyle promote a similar increase in oxidative stress and locomotor muscle atrophy?
- **What is the main finding and its importance?**
  Prolonged skeletal muscle inactivity causes both oxidative stress and muscle atrophy. The findings provide the first evidence that the transition from an active to a sedentary lifestyle rapidly leads to impaired antioxidant defence and increased oxidative stress in load‐bearing skeletal muscles, resulting in the onset of atrophy.

## 1 INTRODUCTION

Prolonged periods of skeletal muscle disuse result in the rapid development of oxidative damage in muscle fibres and the associated loss of muscle protein, resulting in muscle weakness (Powers et al., 2020). Although the mechanism(s) responsible for disuse muscle atrophy in humans remains an active area of research, the mechanisms responsible for inactivity‐induced muscle atrophy have been mechanistically investigated in preclinical studies (reviewed in Michel et al., 2025; Powers & Schrager, 2022). Specifically, prolonged inactivity in rodent skeletal muscle is associated with dysregulated proteostasis due to both accelerated proteolysis and depressed protein synthesis (Ozdemir et al., 2022; Powers et al., 2020). This inactivity‐induced impairment in muscle health is significant because skeletal muscle is a key endocrine organ that plays an important role in regulating metabolic homeostasis (Karstoft & Pedersen, 2016; Pedersen, 2009, 2010). Indeed, physical inactivity is linked to numerous chronic diseases, including type 2 diabetes (Booth et al., 2012).

To date, most studies investigating the impact of skeletal muscle inactivity on muscle fibre size and function have employed preclinical models of muscle disuse, including denervation, limb immobilization and hindlimb unloading. Each of these models of skeletal muscle disuse confirms that prolonged muscle disuse promotes the rapid development of oxidative stress and muscle atrophy (Min et al., 2011; Muller et al., 2007; Nuoc et al., 2017; O'Leary & Hood, 2009; Powers et al., 2005). Although each of these experimental models has clinical relevance, none of these paradigms mimic the impact that physical inactivity or a sedentary lifestyle has on skeletal muscles. In this regard, it is important to appreciate that physical inactivity and sedentary lifestyle are not the same. Specifically, physical inactivity refers to individuals who do not perform the recommended amount of physical activity. In contrast, a sedentary lifestyle describes individuals that remain sedentary for more than 6 h per day (Chuang et al., 2024; Sedentary Behaviour Research, 2012). Sedentary behaviour is defined as waking behaviours that involve low energy expenditure (i.e., <1.5 METs); common sedentary behaviours include sitting, reclining and lying (Pate et al., 2008; Sedentary Behaviour Research, 2012). Expressed in MET‐hours, a sedentary lifestyle is defined as someone who engages in 9 MET‐hours per day of inactivity (6 × 1.5 = 9 MET‐hour per day). Importantly, a sedentary lifestyle is also associated with an increased risk of developing several chronic diseases (Cao et al., 2022; Chuang et al., 2024; Ekelund et al., 2019; Hamilton et al., 2014; Jingjie et al., 2022). Therefore, it is important to investigate the impact of sedentary behaviour on skeletal muscle health.

Recently, a novel preclinical model of sedentary behaviour for rodents has emerged that permits the study of the effect of a sedentary lifestyle on skeletal muscle health (Alabi & Akomolafe, 2022; Marmonti et al., 2017). This model confines animals in small cages that permit easy access to food and water but limits physical activity to encourage sedentary behaviour. Recent studies concluded that this model better simulates sedentary behaviour in humans compared to previous models of extreme muscle disuse (Alabi & Akomolafe, 2022). Therefore, this new preclinical model provides an opportunity to investigate the impact of a sedentary lifestyle on oxidative damage and atrophy of skeletal muscle fibres. Thus, to determine the effect of a sedentary lifestyle on skeletal muscle, we tested the hypothesis that the transition from active to sedentary living results in the rapid development of oxidative damage and reduced skeletal muscle mass in rodent load‐bearing skeletal muscles.

## 2 METHODS

### 2.1 Ethical approval

The experimental protocol complied with the ‘Regulation on the Working Procedures and Principles of Animal Experimentation Ethics Committees’ numbered 28914 issued by the Ministry of Forestry and Water Affairs of Turkey in 2014. The study was conducted with the approval of Hacettepe University Animal Experiments Local Ethics Committee (Approval No. 2022/08‐07)

### 2.2 Animal care

Sixteen 5‐month‐old male Wistar albino rats weighing 300–350 g were procured from the Experimental Animals Laboratory Industry and Trade Company, Ankara, Türkiye. The animals were individually housed in cages under controlled environmental conditions, including ambient temperature (22 ± 2°C), and relative humidity (50 ± 5%) and maintained on a regular 12‐h light–dark cycle starting at 06.00 h.

### 2.3 Experimental protocol

**FIGURE 1:**
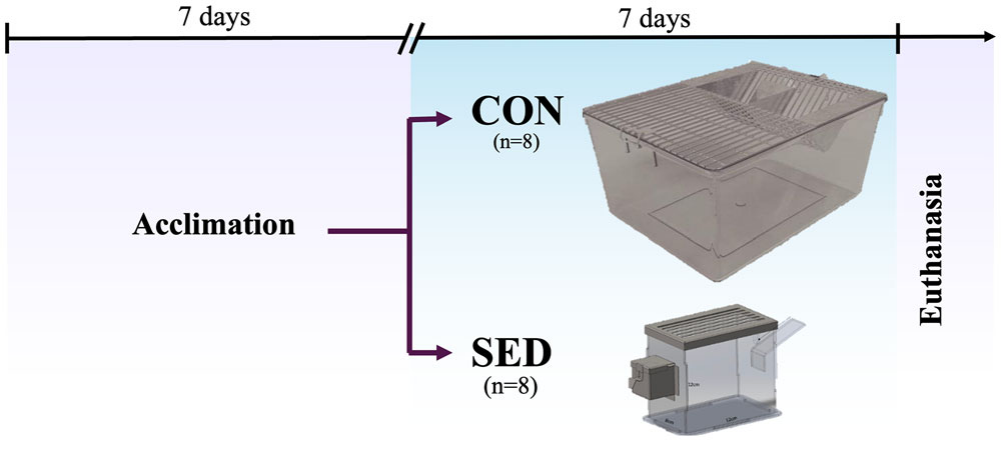
Experimental design. Following acclimation, rats were randomly assigned to CON or SED group. After 7 days of the experimental protocol, rats were sacrificed on day 8.

After arrival at the facility, animals acclimatized to the laboratory conditions for 1 week before the initiation of experiments to minimize the impact of the transport stress. Animals were randomly assigned into control (CON, *n* = 8) or sedentary (SED, *n* = 8) groups following acclimation. The SED group animals were kept for seven consecutive days in reduced‐volume cages. Both the control and experimental animals were housed individually (Figure 1). A standard maintenance rat diet and water were provided ad libitum. The animals’ body weight and food intake were monitored daily during the experimental period.

### 2.4 Induction of sedentary life

**FIGURE 2:**
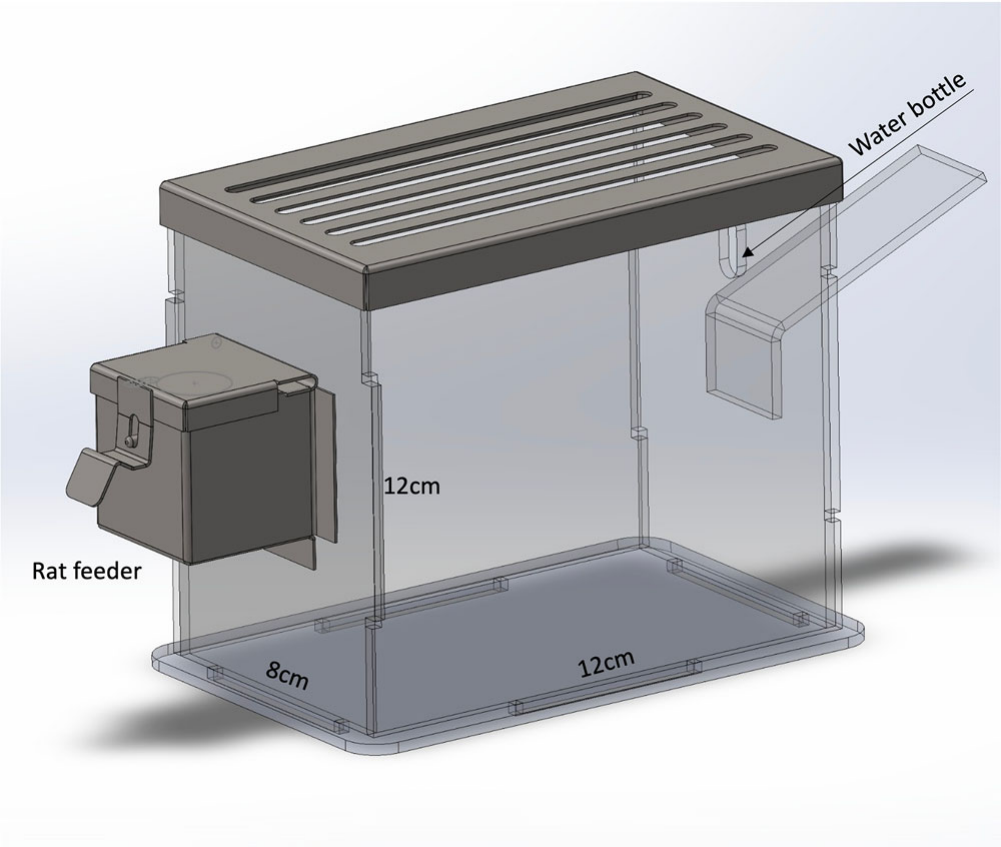
Representative picture of sedentary lifestyle model based on cage volume reduction. This model allows animals to remain immobilized, mimicking reduced physical activity conditions in humans in almost all aspects such as bed rest and sedentary lifestyle. Group SED animals were housed in small Plexiglas cages with 12 × 12 × 8 cm dimensions instead of standard rat cage.

In the current study, we employed the model originally introduced by Marmonti et al. (2017), incorporating several modifications. Briefly, small Plexiglas cages with 12 × 12 × 8 cm dimensions were designed (Figure 2). These reduced‐volume cages corresponded to approximately 20% of standard rat cages, allowing the animal to move and have access to food and water. There were grilles on the ceiling of the cage, providing air circulation for the animal to breathe comfortably. Cage cleaning and bedding changes were performed twice a day during the whole immobilization period by transferring the animals to pre‐prepared cages of the same size to prevent free movement of the animals. The activity levels of animals housed in the reduced‐volume cage remained similar throughout the 7‐day period.

### 2.5 Tissue removal and preparation

The rats were sacrificed through exsanguination on day 8 under deep anaesthesia by injecting an anaesthetic cocktail solution composed of 90 mg kg^−1^ ketamine and 10 mg kg^−1^ xylazine. After collection of blood samples, serum samples were separated by centrifugation and aliquots were stored at −80 °C for measurement of corticosterone by liquid chromatography−tandem mass spectrometry (LC–MS/MS). Soleus muscles were trimmed from connective tissue and weighed, frozen in liquid nitrogen, and stored at −80°C until biochemical analysis.

### 2.6 Determination of corticosterone levels

Serum corticosterone analyses were performed by ultraperformance liquid chromatography–mass spectrometry (Waters Acquity Triple Quadrupole; Waters, Milford, MA, USA) using the Acquity UPLC BEH C18 column. Mefruside was used as an internal standard. Quantification of corticosterone is performed using selective reaction monitoring of precursors to product ion transitions, 347.2 > 329.2 *m*/*z*.

### 2.7 Advanced oxidation protein product content

Advanced oxidation protein products (AOPPs) are markers of oxidative stress and protein modification. AOPPs are formed as a result of the reaction of chlorine‐based oxidants such as hypochlorous acid and chloramines with proteins (Witko‐Sarsat et al., 2003). AOPPs were determined by the spectrophotometric method as described by Witko‐Sarsat et al. (1996). Briefly, 30 mg soleus muscle was homogenized on ice in a buffer containing 5 mM Tris–HCl, 5 mM EDTA, pH 7.4. and protease inhibitors. Homogenates were centrifuged for 10 min at 1500 *g*, 4°C. Samples were prepared by diluting supernatants 1:8 in phosphate buffered saline. Ninety‐six‐well microtitre plates were loaded with 200 µL of the sample, followed by 10 µL 1.16 M potassium iodide (KI). After incubation with KI for 2 min, the reaction was stopped by adding 20 µL glacial acetic acid. The absorbance of the reaction mixture was immediately read at 340 nm in a microplate reader. A chloramine T (0–100 µM) standard curve was used to determine the AOPP content in the samples. The concentration of AOPPs was normalized to the total protein content determined by the bicinchoninic acid (BCA) assay (Takara Bio Inc., Shiga, Japan).

### 2.8 Western blotting

Fifty milligram of soleus was homogenized with a buffer (5 mM Tris–HCl, 5 mM EDTA, pH, 7.4) containing protease inhibitors. Homogenates were centrifugated at 1500 *g* for 10 min at 4°C before determining protein concentration by BCA assay (Takara Bio Inc.). Levels of 4‐HNE‐conjugated proteins were determined by western blot. Briefly, 33 µg of protein was loaded into SDS‐PAGE gel. SDS‐PAGE was performed on 12% gels prepared with an acrylamide:bisacrylamide ratio of 37.5:1. Proteins were separated according to their molecular weights by electrophoresis system (Bio‐Rad Laboratories, Hercules, CA, USA) for approximately 2 h. Then, the proteins were transferred to 0.45 µm‐thick nitrocellulose membranes (Bio‐Rad) with a semi‐dry transfer system (Trans‐Blot Turbo, Bio‐Rad) at 25 V electric current for 30 min. After the transfer process, the membranes were washed with Tris‐buffered saline with 0.1% Tween 20 (TBST) and subsequently blocked with TBST containing 5% dry milk for 1 h at room temperature. Then, the membranes were incubated overnight with 4‐HNE specific primary antibody (Abcam, Camb, cat. no. ab46545) at 4°C. After 1 h of incubation with anti‐rabbit IgG, horseradish peroxidase‐conjugated secondary antibody (Cell Signaling Technology, Danvers, MA, USA, cat. no. 7074), protein bands were visualized by an enhanced chemiluminescence substrate (Bio‐Rad, cat. no. 170‐5061). Then, 4‐HNE‐conjugated protein bands between 100 and 25 kDa were visualized by the chemiluminescence imaging system (Chemi‐Doc, Bio‐Rad). Protein bands were normalized to total protein obtained by Ponceau S staining of membranes.

### 2.9 Antioxidant enzyme activities

To determine the activities of catalase (CAT), glutathione peroxidase (GPX) and superoxide dismutase (SOD), 30 mg of soleus muscle was homogenized in 0.9% NaCl containing 10 mM phosphate buffer, pH 7.4. The homogenates were centrifuged at 10,000 *g* for 10 min at 4°C. The supernatants were used to determine antioxidant enzyme activities, and protein concentration of supernatants was measured by BCA assay (Takara Bio Inc.) for normalization.

GPX, SOD and CAT activities were evaluated by commercial kits (Elabscience, Wuhan, China, cat. no. E‐BC‐K019‐M, E‐BC‐K031‐M, E‐BC‐K096‐M, respectively) according to the manufacturer's instructions. The activity of GPX was assessed through the measurement of reduced glutathione consumption. A stable yellow colour, resulting from the reaction between reduced glutathione and dinitrobenzoic acid, was quantified by determining the absorbance at 412 nm. SOD activity was determined based on the inhibition of superoxide anions (O_2_^−^ **^.^**) in the supernatant and was calculated by measuring the purple colour formed by the reaction of nitrite with the chromogenic agent at 550 nm. Catalase activity was assessed by measuring the complex formed from the reaction of H_2_O_2_ with ammonium molybdate at 405 nm, which decreased with the addition of the supernatant.

### 2.10 Statistical analysis

Data for all groups are presented as the mean ± SD. Data normality was verified by the Shapiro–Wilk test. All data were evaluated by an independent samples Student's *t*‐test. To examine the relationships between the oxidative products (AOPPs and 4‐HNE) and soleus weight to body weight ratio, Pearson's correlation analysis was conducted. Statistical significance was set at *P* < 0.05.

## 3 RESULTS

This study aimed to investigate oxidative stress in skeletal muscle as a consequence of reduced physical activity, where neural stimulation and mechanical loading on skeletal muscles are reduced. The effect of a sedentary lifestyle on body weight, daily food and water intake are shown in Table 1. The daily water consumption of animals subjected to the small cage was not significantly different from the control group (*P *= 0.734). Living in a reduced‐volume cage for 7 days seems to decrease food intake, possibly as a result of reduced energy expenditure (*P = *0.055).

**TABLE 1:** The effect of sedentary life on body weight, soleus weight, food and water intake.

	CON (*n* = 8)	SED (*n* = 8)
Initial body weight (g)	344.0 ± 16.5	337.4 ± 14.8
Final body weight (g)	370.0 ± 15.0	353.4 ± 15.7
Soleus weight (mg)	146 ± 11.6	115.4 ± 6.6*
Food intake (g day^−1^)	26.0 ± 2.8	23.6 ± 1.1
Water intake (mL day^−1^)	44.3 ± 8.5	45.7 ± 7.0

Data are reported as means ± SD. Statistical significance was tested by independent samples Student's *t*‐test (*P < 0.001).

Table 2 shows the adrenal gland weights and serum corticosterone levels of the animals, and the effects of living in a reduced‐volume cage for 7 days on adrenal gland weight/body weight (*P *= 0.574) and serum corticosterone levels (*P *= 0.057).

**TABLE 2:** Serum corticosterone levels and adrenal glands weight.

	CON (*n* = 8)	SED (*n* = 8)
Corticosterone (ng mL^−1^)	11.2 ± 2.4	8.6 ± 2.7
Adrenal glands weight/body weight (mg g^−1^)	0.15 ± 0.02	0.14 ± 0.03

Data are reported as means ± SD. Statistical significance was tested by independent samples Student's *t*‐test.

**FIGURE 3:**
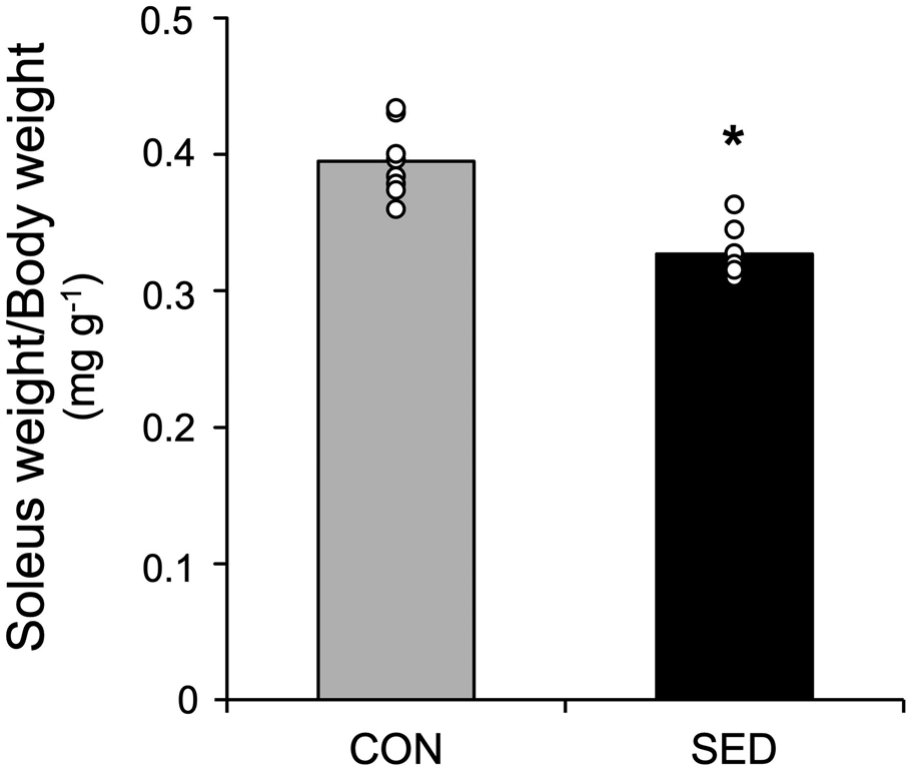
The effect of 7 days of a sedentary life on soleus weight/body weight ratio. Statistical difference was tested by independent samples Student's *t*‐test. *P < 0.001 versus CON.

To determine whether immobilization causes skeletal muscle atrophy, soleus weight was measured and expressed as the soleus weight/body weight ratio (Figure 3). The rats housed in reduced‐volume cages had 17.2% lower soleus weight/body weight compared to the control group (*P *< 0.001).

**FIGURE 4:**
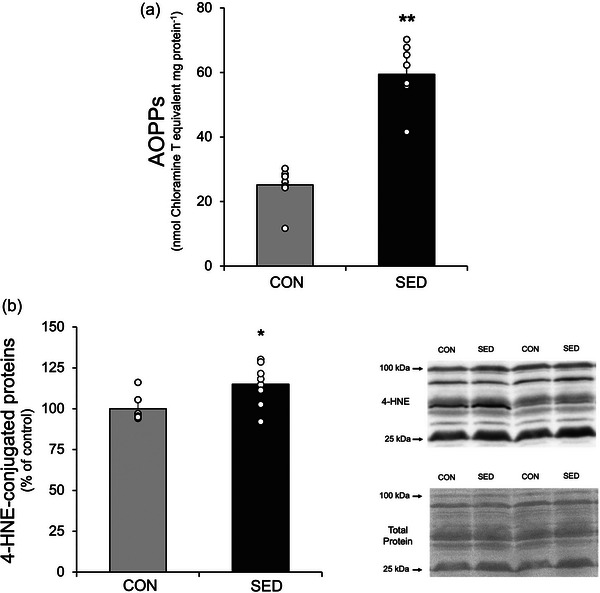
The effect of 7 days of a sedentary life on oxidative damage in soleus. (a) AOPPs level. (b) The optical density units of 4‐HNE‐conjugated proteins in soleus and representative western blot for 4‐HNE‐conjugated proteins between 100 and 25 kDa and total protein normalization with ponceau S staining. Statistical difference was tested by independent samples Student's *t*‐test. *P < 0.05, **P < 0.001 versus CON.

To investigate the effect of a sedentary lifestyle on oxidative damage in soleus muscles, AOPPs level was analysed by a spectrophotometric method as an indicator of protein oxidation. The levels of the lipid peroxidation marker 4‐hydroxynonenal (4‐HNE) analysed by western blot are shown in Figure 4.

Sedentary lifestyle resulted in a more than two‐fold increase in the AOPP level in soleus muscles (Figure 4a). In addition, 7 days of sedentariness increased in the 4‐HNE‐conjugated proteins (*P* < 0.05) (Figure 4b).

**FIGURE 5:**
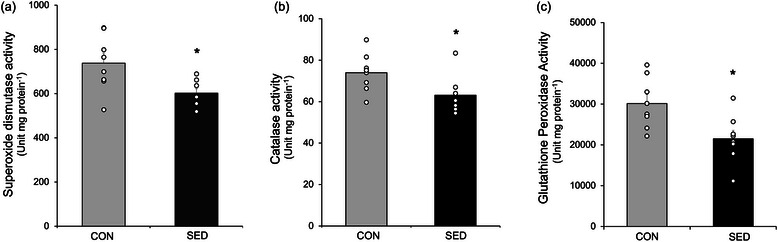
The effect of 7 days of sedentary life on antioxidant enzyme activities in soleus. (a) Superoxide dismutase activity. (b) Catalase activity. (c) Glutathione peroxidase activity. Statistical difference was tested by independent samples Student's *t*‐test. *P < 0.05 versus CON.

To assess the impact of sedentary lifestyle on the antioxidant defence system in soleus, SOD, CAT and GPX activities were analysed by a spectrophotometric method (Figure 5). We showed that 7 days of sedentariness resulted in decreased activity of SOD, CAT and GPX in the soleus (*P *< 0.05) (Figure 5).

**FIGURE 6:**
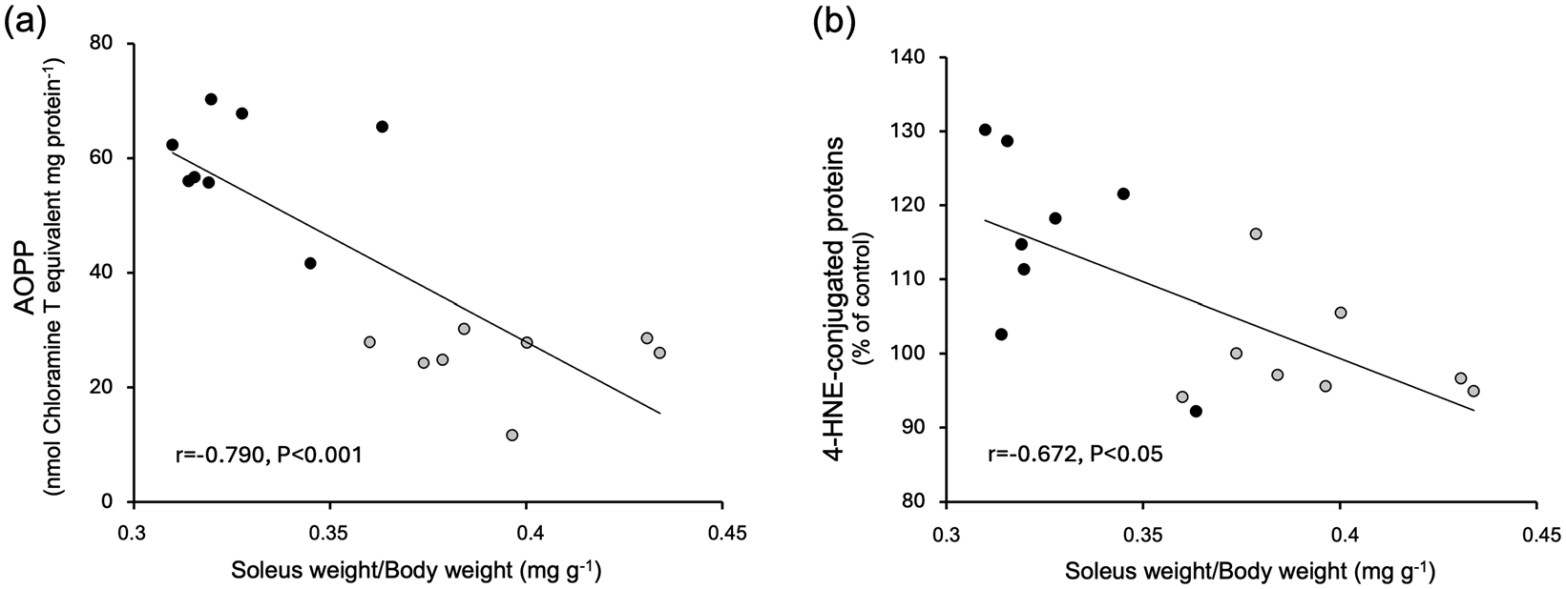
Linear relationship between oxidative stress and soleus muscle loss. (a) Soleus AOPPs level and relative muscle weight. (b) Soleus 4‐HNE‐conjugated protein levels and relative muscle weight. Gray circles, CON (*n* = 8); black circles, SED (*n* = 8).

Figure 6a and b shows that both AOPPs and relative 4‐HNE‐conjugated protein levels were inversely correlated with the soleus to body weight ratio (*r* = −0.790, *P *< 0.001 and *r* = −0.672, *P *< 0.05, respectively).

## 4 DISCUSSION

### 4.1 Overview of major findings

To investigate the effect that a transition from an active to a sedentary lifestyle has on skeletal muscle, we employed a novel preclinical model that mimics sedentary behaviour in humans. Importantly, our results provide the first evidence that the transition from active to sedentary living promotes the rapid development of lipid peroxidation and a loss of fibre mass in weight‐bearing skeletal muscles. Indeed, our findings reveal that as little as 7 days of a sedentary lifestyle results in ∼17% reduction in the soleus muscle weight to body weight ratio. Notably, this sedentary living induction of muscle atrophy was associated with increased oxidative stress as evidenced by the high levels of oxidative damage biomarkers (i.e., AOPPs and 4‐HNE). The inverse correlation between markers of oxidative stress (AOPPs and relative 4‐HNE levels) and the soleus‐to‐body weight ratio suggests that oxidative stress induced by a sedentary lifestyle may be associated with load‐bearing muscle loss. Finally, our data also suggest that the sedentary lifestyle‐induced oxidative stress in muscle may be the result of a decrease in antioxidant enzyme activity (i.e., SOD, GPX and CAT) in the muscle fibres. A brief discussion of each of these key findings follows.

### 4.2 Sedentary lifestyle promotes skeletal muscle atrophy

Proteostasis results from a balance between the rates of protein synthesis and protein breakdown and proteostasis is essential to maintaining muscle health (Powers, Wiggs et al., 2012). Specifically, protein synthesis minus protein breakdown determines the net protein balance in muscle fibres. In this regard, numerous rodent studies using a variety of models of muscle disuse have concluded that inactivity‐induced loss of muscle protein (i.e., atrophy) occurs due to both a decrease in muscle protein synthesis and accelerated proteolysis (Atherton et al., 2016; Bodine, 2013; Krawiec et al., 2005).

The current study demonstrates, for the first time, that the transition from an active lifestyle to a sedentary lifestyle is associated with a rapid rate of load‐bearing muscle atrophy. Indeed, only 7 days of a sedentary lifestyle resulted in an ∼17% decrease in the soleus muscle/body weight ratio. This remarkable rate of muscle atrophy is only slightly lower than the rate of muscle atrophy observed in limb muscle disuse resulting from casting (Vazeille et al., 2008). Therefore, our findings confirm that transitioning from an active life to a sedentary behaviour promotes a rapid and significant level of limb muscle atrophy.

### 4.3 Transition to a sedentary lifestyle promotes rapid increases in oxidative damage to skeletal muscle fibres

Studies during the past three decades have documented that disuse muscle atrophy is associated with increased oxidative stress in muscle fibres (Min et al., 2011; Muller et al., 2007; Nuoc et al., 2017; O'Leary & Hood, 2009; Powers et al., 2005). Although inactivity‐induced radical production likely occurs at several locations in the muscle fibre, evidence reveals that mitochondria are a dominant source of radical production in skeletal muscles exposed to prolonged periods of inactivity (Powers, Wiggs et al., 2012). Moreover, previous work in diaphragm muscle reveals that prolonged diaphragmatic inactivity during mechanical ventilation results in a diminished total antioxidant capacity (Falk et al., 2006). Similarly, the current experiments provide the first evidence that sedentary behaviour results in a decrease in the activity of three key antioxidant enzymes in limb muscles. Specifically, our results reveal that only 7 days of sedentary living results in significant decreases in the activities of SOD, GPX and CAT. This observation is important because SOD is responsible for dismutation of the superoxide radical into the non‐radical oxidant, hydrogen peroxide (H_2_O_2_) (Fridovich, 1997). Further, GPX and CAT play important roles in eliminating H_2_O_2_ from the cell to prevent oxidation of proteins and lipids. Together, these changes in key antioxidant enzymes reveal that sedentary living has a negative impact on the muscle antioxidant capacity.

This sedentary living‐induced decrease in the total antioxidant capacity in muscle fibres can significantly impact muscle atrophy. Indeed, evidence reveals that oxidant‐mediated redox signalling influences signalling pathways that regulate both protein synthesis and proteolysis in skeletal muscle fibres (Powers & Schrager, 2022; Powers et al., 2020, 2011; Sartori et al., 2021). In particular, inactivity‐induced oxidative stress promotes a decrease in muscle protein synthesis and increases muscle proteolysis (Powers, Smuder et al., 2012). The increased muscle protein degradation is mediated by oxidant‐induced activation of all four major proteolytic systems including calpain, the ubiquitin–proteasome system, autophagy and caspases (Cohen et al., 2015; Egerman & Glass, 2014; Powers et al., 2016). Nonetheless, our measurements of muscle atrophy do not reveal whether proteolysis or depressed protein synthesis played the dominant role in the observed loss of muscle mass.

### 4.4 Critique of experimental model

Most of our knowledge about inactivity‐induced muscle wasting in load‐bearing muscles has been garnered from rodent studies of locomotor muscle disuse using limb immobilization (e.g., casting) or hindlimb suspension. Further, studies of respiratory muscle inactivity (e.g., diaphragm) using mechanical ventilation have provided unique insight into the mechanisms responsible for inactivity‐induced muscle atrophy (Falk et al., 2006; Kavazis et al., 2009; Shanely et al., 2002). Although these approaches to studying muscle atrophy have clinical relevance, these models of muscle disuse do not reflect the levels of muscle inactivity observed in humans living a sedentary lifestyle (Reidy et al., 2021)

In the current experiments using rats, we used a small‐cage model designed to mimic high levels of sedentary behaviour in humans. This preclinical model of sedentary behaviour permits the study of the effect of a sedentary lifestyle on skeletal muscle health (Alabi & Akomolafe, 2022; Marmonti et al., 2017; Reidy et al., 2021; Roemers et al., 2019; Siripoksup et al., 2024). Specifically, this model confines animals in small cages that permit free access to food and water but limits physical activity to promote sedentary behaviour. A recent review concludes that, compared to previous models of extreme muscle disuse, the small‐cage model better simulates sedentary behaviour in humans (Reidy et al., 2021); this conclusion has been supported by others (Alabi & Akomolafe, 2022; Roemers et al., 2019; Siripoksup et al., 2024). Finally, similar to the findings of a previous study (Marmonti et al., 2017), the absence of increases in serum corticosterone concentrations and adrenal gland weights suggests that living in a reduced‐volume cage for 7 days does not induce a stress response.

### 4.5 Conclusions and future directions

To our knowledge, these are the first preclinical experiments to reveal that the transition from an active lifestyle to a sedentary lifestyle result in the rapid onset of both oxidative damage and atrophy in limb muscles. This is an important new finding that provides a warning about the negative impact that a sedentary lifestyle has on skeletal muscle health.

In regard to a sedentary lifestyle and muscle health, many unanswered questions remain. For example, can the negative effects of prolonged sedentary behaviour be countered with short periods of intermittent exercise scattered throughout the day? Further, do regular bouts of exercise training result in a preconditioning of muscle fibres that protects against the atrophy and oxidative damage associated with prolonged sedentary behaviour? Also, are mitochondrial‐targeted antioxidants a potential countermeasure to protect skeletal muscle against sedentary behaviour‐induced muscle atrophy and oxidative stress? Clearly, there is much more to be learned about this important topic.

## AUTHOR CONTRIBUTIONS

Conception or design of the work: I.G.O., S.A., S.P., H.D.; acquisition or analysis or interpretation of data for the work: I.G.O., S.A., S.O.B., H.D.; drafting the work or revising it critically for important intellectual content: I.G.O., S.A., S.P., S.O.B., H.D. All authors have read and approved the final version of this manuscript and agree to be accountable for all aspects of the work in ensuring that questions related to the accuracy or integrity of any part of the work are appropriately investigated and resolved. All persons designated as authors qualify for authorship, and all those who qualify for authorship are listed.

## CONFLICT OF INTEREST

None declared.

## Data Availability

The datasets generated during and/or analysed during the current study are available from the corresponding author upon reasonable request.
